# Increased Reward-Related Activation in the Ventral Striatum During Stress Exposure Associated With Positive Affect in the Daily Life of Young Adults With a Family History of Depression. Preliminary Findings

**DOI:** 10.3389/fpsyt.2020.563475

**Published:** 2021-01-18

**Authors:** Chantal Martin-Soelch, Matthias Guillod, Claudie Gaillard, Romina Evelyn Recabarren, Andrea Federspiel, Christoph Mueller-Pfeiffer, Philipp Homan, Gregor Hasler, Dominik Schoebi, Antje Horsch, Patrick Gomez

**Affiliations:** ^1^IReach Lab, Unit of Clinical and Health Psychology, Department of Psychology, University of Fribourg, Fribourg, Switzerland; ^2^Section on Neurobiology of Fear and Anxiety, National Institutes of Mental Health, Bethesda, MD, United States; ^3^Translational Research Center, University Hospital of Psychiatry and Psychotherapy, Bern, Switzerland; ^4^Department of Consultation-Liaison-Psychiatry and Psychosomatic Medicine, University Hospital Zurich, University of Zurich, Zurich, Switzerland; ^5^Center for Psychiatric Neuroscience, Feinstein Institute for Medical Research, Manhasset, NY, United States; ^6^Division of Psychiatry Research, Zucker Hillside Hospital, Northwell Health, New York, NY, United States; ^7^Department of Psychiatry, Zucker School of Medicine at Northwell/Hofstra, Hempstead, NY, United States; ^8^Unit of Psychiatry Research, University of Fribourg, Fribourg, Switzerland; ^9^Unit of Clinical Family Psychology, Department of Psychology, University of Fribourg, Fribourg, Switzerland; ^10^Department Woman-Mother-Child, Lausanne University Hospital, Lausanne, Switzerland; ^11^Institute of Higher Education and Research in Healthcare, University of Lausanne, Lausanne, Switzerland; ^12^Center for Primary Care and Public Health (Unisanté), University of Lausanne, Lausanne, Switzerland

**Keywords:** depression, reward, striatum, stress, positive affect (PA), negative affect (NA), ambulatory assessment (AA), fMRI

## Abstract

**Background:** Being the offspring of a parent with major depression disorder (MDD) is a strong predictor for developing MDD. Blunted striatal responses to reward were identified in individuals with MDD and in asymptomatic individuals with family history of depression (FHD). Stress is a major etiological factor for MDD and was also reported to reduce the striatal responses to reward. The stress-reward interactions in FHD individuals has not been explored yet. Extending neuroimaging results into daily-life experience, self-reported ambulatory measures of positive affect (PA) were shown to be associated with striatal activation during reward processing. A reduction of self-reported PA in daily life is consistently reported in individuals with current MDD. Here, we aimed to test (1) whether increased family risk of depression is associated with blunted neural and self-reported reward responses. (2) the stress-reward interactions at the neural level. We expected a stronger reduction of reward-related striatal activation under stress in FHD individuals compared to HC. (3) the associations between fMRI and daily life self-reported data on reward and stress experiences, with a specific interest in the striatum as a crucial region for reward processing.

**Method:** Participants were 16 asymptomatic young adults with FHD and 16 controls (HC). They performed the Fribourg Reward Task with and without stress induction, using event-related fMRI. We conducted whole-brain analyses comparing the two groups for the main effect of reward (rewarded > not-rewarded) during reward feedback in control (no-stress) and stress conditions. Beta weights extracted from significant activation in this contrast were correlated with self-reported PA and negative affect (NA) assessed over 1 week.

**Results:** Under stress induction, the reward-related activation in the ventral striatum (VS) was higher in the FHD group than in the HC group. Unexpectedly, we did not find significant group differences in the self-reported daily life PA measures. During stress induction, VS reward-related activation correlated positively with PA in both groups and negatively with NA in the HC group.

**Conclusion:** As expected, our results indicate that increased family risk of depression was associated with specific striatum reactivity to reward in a stress condition, and support previous findings that ventral striatal reward-related response is associated with PA. A new unexpected finding is the negative association between NA and reward-related ventral striatal activation in the HC group.

## Introduction

Major depression disorder (MDD) is a leading cause of disability worldwide, and a research priority in mental health. Having a family history of depression (FHD) is a strong and consistent predictor of MDD development (1–3). In particular, the offspring of parents with MDD have a higher probability of experiencing poorer physical, psychological, or social health (4), as well as a two- to five-fold increased risk of experiencing an episode of MDD, and an increased risk of earlier onset of MDD (i.e., adolescence) (5).

Anhedonia, i.e., the reduced ability to enjoy once-pleasurable activities is a core feature of MDD (6) that could be partially explained by blunted responses to reward at neural level (7–9). Neural responses to reward are processed by a system of cortical and subcortical structures, including among other the striatum, the orbitofrontal and medio-prefrontal cortex as well the anterior cingulate gyrus, with the striatum, in particular the ventral striatum, being one crucial region involved in the anticipation, consumption, and learning from rewarding stimuli (10–14). The term ventral striatum was coined by Heimer (15) and encompasses the continuity between the nucleus accumbens and the ventral part of putamen and of the ventral caudate as well as rostral internal capsule, the olfactory tubercle and the rostrolateral part of the lateral olfactory tract in primates. In the context of reward, the ventral striatum includes the nucleus accumbens, the medial/ventral caudate nucleus, and the medial and ventral putamen (16). A large number of neuroimaging studies reported that individuals with MDD exhibit reduced reward-related activity in the ventral striatum (VS) (17–20). Interestingly, a similar reduced VS activity in response to reward was also found in individuals with FHD before they have met the criteria for a first episode of MDD (21–24). For instance, reduced striatal activation in response to monetary rewards was evidenced in asymptomatic adolescents and children of parents with MDD compared to age- and gender-matched control groups without FHD (25, 26). Thus, blunted striatal response to reward has been postulated to be a potential endophenotype related to MDD (27).

A growing amount of evidence indicates that stress exposure and stress sensitivity are strongly associated with the onset of MDD (28–32). Stress experiences have been shown to affect striatal reward processing in the context of early-life stress, childhood emotional neglect (33, 34), recent life stress (35), and experimental acute stress (36–38). In most cases, stress experiences reduced the activation of the striatum in response to reward. It has been hypothesized that an imbalance between stress and reward reactivity could be a predictor for the development of psychopathology in general (39, 40) and for MDD in particular (9). In line with that hypothesis, a recent study indicated that reward responsiveness measured with event-related potential had a moderator effect on the relationship between life-stress exposure and depressive symptoms in a large sample of young adults (41). Further findings showed that higher VS response to reward was associated with more reported positive affect (PA) in daily life (21, 35, 42), and supporting evidence suggests that this association could buffer the effect of stress sensitivity [e.g., (43, 44)].

Combined findings from daily life measures and neuroimaging techniques, including functional Magnetic Resonance Imaging (fMRI) and positron emission tomography (PET scan) support the idea that dopaminergic activity in VS related to reward response is associated with self-reported PA in daily life (21, 45, 46). The experience sampling method (ESM) is used to collect self-report measures at multiple points in time in natural settings. It offers the opportunity to capture daily life dynamics related to cognitive and affective experiences, including in individuals with MDD (47–49). PA and negative affect (NA)are traits related to the propensity to experience positive (e.g., happy, confident, joyful) or negative (e.g., sad, angry, ashamed, anxious, lonely) affective states (50) and can be measured with the ESM. PA and NA have been analyzed as both, predictors and outcomes of mental health status (51, 52). Whereas, NA is commonly experienced in almost every mental health disorder (52), there has been an increasing interest in PA in terms of both, its role in daily life and the neuroscientific understanding of psychopathology development and treatment, notably in MDD (21, 45, 51, 53–56). In that context, Forbes et al. (21) showed that reduced reward-related striatal response in adolescents with MDD compared to healthy participants was associated with lower subjective PA in everyday life. In addition, the frequency of reported PA has been conceptualized as an indicator of reward reactivity in daily life (57). Therefore, recording PA in daily life in association with neural measures of reward and stress seems a promising way to investigate the effects of the stress-reward interaction on the development of MDD symptoms, in particular in vulnerable individuals. To our knowledge, one study has examined first-degree relatives of individuals with psychotic disorders (58), but none has investigated first-degree relatives of individuals with MDD.

Based on the above considerations, we propose here an innovative way to investigate the complexity of family risk of MDD by combining neuroimaging measures of reward processing with everyday life reward-related measures, using an ESM protocol in association with fMRI measurements. The aims of this study were: (1) To investigate whether increased family risk of depression is associated with blunted neural and self-reported reward responses. We expected lower neural and self-reported reward sensitivity in individuals with FHD in comparison to healthy controls (HC). (2) To test the stress-reward interactions at the neural level. We expected a stronger reduction of reward-related striatal activation under stress in FHD individuals compared to HC. (3) To explore associations between fMRI and daily life self-reported data on reward and stress experiences, with a specific interest in the striatum as a crucial region for reward processing. Based on the results of (21), we expected positive correlations between PA and reward-related striatal activation to be more accentuated in HC than in FHD participants as well as negative correlations with NA and self-reported stress that would be more accentuated in the FHD group than in the HC group. We focused here on the striatum, in particular the VS, because (1) it is a crucial region in all phases of reward processing (12), (2) it is a region in which differences were reported in the reward-related neural activation between depressed and not-depressed participants (17, 19) as well as between individuals with a family history of depression and controls (22, 23), and (3) this region was reported to be correlated with positive emotions in everyday life (45) #147. We focused on the reward-related activation during the outcome phase, because a recent meta-analysis indicated that differences in the reward-related striatal activation between depressed and control participants were mostly measured activation during the outcome phase (or reward delivery phase) (59) and because robust striatal differences between FHD and healthy participants have been evidenced in this phase in particular (27).

## Materials and Methods

### Participants

Sixteen asymptomatic first-degree relatives with family history of MDD (FHD; 12 females, mean age = 24.31 years, SD = 4.08), and sixteen age-, gender- and socioeconomic status (SES)-matched healthy controls (HC; 12 females, mean age = 25.19 years, SD = 4.79) with no parental history of mental disorder were recruited from the local community by advertisement at the University of Fribourg. The participants of the control group were selected from a larger sample [see (36)] to match for age and gender the group of participants with increased family risk of depression. Participation was compensated in money and/or experimental hours for study plans. The inclusion criteria were: age between 18 and 40 years; good health; good understanding of French; compliance with study procedure; and, for the FHD group, having a first-degree relative with a diagnosed major depressive disorder (MDD), or, for HC group, having no mental health history, as assessed with the Family interview for Genetic Studies (FIGS) (60). General exclusion criteria were: current or past history of any mental disorder, as determined by the Mini International Neuropsychiatric Interview (MINI) (61); history of any endocrinological conditions; history of any neurological condition, epilepsy or head injury; use of psychoactive substances, including alcohol (CAGE) (62), tobacco (Fagerström Test for Nicotine Dependence) (63), and cannabis (CAST) (64); being at risk for pathological gambling (Lie/bet) (65); non-removable metal elements in or on the body; pregnancy, which was confirmed by a urine test on the day of the scan; and being left-handed, as determined with the Edinburgh Handedness Inventory—short form (EHI) (66). Participants were mainly university students (FHD; 87%, HC; 81%) from the Swiss middle-class population. Table 1 shows that groups did not differ significantly in socioeconomic status (SES). Depressive symptoms were assessed with the Beck depression inventory II (BDI-II) (69), and the Montgomery and Asberg depression rating scale (MADRS) (68), and state and trait anxiety were assessed with the Spielberger State-Trait Anxiety Inventory (STAI) (70). This study was approved by the local ethical review boards of Vaud and Fribourg region (Commission cantonale d'éthique de la recherche sur l'être humain (CER-VD), Study Number 261/14) as well as that of the Bern region (Kantonale Ethikkommission Bern (KEK BE), Study Number 337/14). All participants provided written informed consent that conformed to the guidelines set out in the Declaration of Helsinki (2013).

**Table 1:** Descriptive statistics and comparison analyses between family history of depression and healthy control groups.

	**FHD *N* = 16**	**HC*N* = 16**	**Test *t;* χ^2^**
	**Mean (S.D.) [Range]**	**Mean (S.D.) [Range]**	**df = (30; 1)**
**Sociodemographic information**			
Sex: female, *n*	12 (75%)	12 (75%)	0, *p* = 1
Age	24.31 (4.08) [20–36]	25.19 (4.79), [20–37]	−0.56, *p* = 0.582
SES	58.19 (17.14) [23–77]	58.06 (16.69), [15–84]	−0.02, *p* = 0.983
Students: *n*	14 (87.5%)	13 (81.3%)	0.24, *p* = 0.626
**Parent with MDD history**			
Mother, *n*	11 (69%)		
Father, *n*	4 (25%)		
Both, *n*	1 (6%)		
Age at parental MDD onset	12.56 (7.75) [0–25]		
Having lived with MDD parent, *n*	15 (94%)		
Currently living with MDD parent, *n*	7 (44%)		
Clinical information: [range]			
MADRS [0–60]	3.81 (2.81) [0–9]	4.37 (4.42) [0–14]	−0.43, *p* = 0.671
STAI A	30.5 (9.25) [20–53]	29.19 (5.75) [20–42]	−0.48, *p* = 0.7
STAI B	31.38 (10.94) [20–60]	34.5 (10.37) [21–53]	−0.82, *p* = 0.96
BDI-II [0–63]	6.69 (6.82) [1–25]	5.8 (5.21) [1–19]	0.38, *p* = 0.708
ESM protocol: [range]			
PA [0–6]	4.22 (0.85) [2.46–5.43]	4.31 (0.84) [3.18–5.9]	−0.30, *p* = 0.764
NA [0–6]	0.87 (0.79) [0.13–2.55]	0.95 (0.71) [0.03–2.38]	−0.32, *p* = 0.748
Subjective stress [0–9]	2.29 (1.49) [0.56–6.57]	2.48 (1.29) [0.36–4.22]	−0.40, *p* = 0.691

*FHD, Family history of depression; HC, Healthy control; MDD, Major depression disorder; SES, Socioeconomic status assessed with the index of socioeconomic position (1–35 lower class; 36–54 lower-middle class; 55–67 middle class; 68–80 upper-middle class; >80 upper class) IPSE; (67); MADRS, semi-structured interview Montgomery-Asberg depression rating scale (68); BDI-II, Beck Depression Inventory II (69); STAI, State Trait Anxiety Inventory (68), A, state; B, trait; ESM, Experience Sampling Method; PA, Positive affect; NA, Negative affect*.

### Procedure

The first meeting included assessment of the inclusion/exclusion criteria. Participants then received detailed explanations of the ESM protocol and we planned the MRI session. ESM material included an iPod 5 Touch (Apple^©^) with the iDialogPad (Mutz^©^) app, for collecting real-time, self-reported data over seven consecutive days (from Monday to Sunday). This decision was made to enable participants to follow the more consistent rhythm of a standard week (71). An alarm was programmed to emit a signal (“beep”) at four precise times during the day: 11:00 a.m. (T1), 2:00 p.m. (T2), 6:00 p.m. (T3), and 9:00 p.m. (T4). Participants self-reported their affective states and subjective stress 30 min after waking in the morning (T0). In most cases, ESM data collection started the week after the initial meeting and the scan session. A final clinical interview was conducted to ensure that participants finished without any outstanding questions or inconveniences related to their participation.

### Measurements

#### ESM Measurements

A total of 1,062 observations were collected, which represents a 95% participant compliance rate. The lowest participation was in 25 self-reported observations (71%), which satisfied the criteria for a representative sample of data (72). Affective states were rated by participants using statements that began with: “At the moment, emotionally I feel….” These were rated on 7-point Likert scales (1 = Not true at all to 7 = Totally true). Items were selected from the PANAS-X (73) and from Wichers et al. (74). We included an additional item, “vulnerable,” to reflect a negative low-dominance affective state. The were “confident” and “happy” for positive affect (PA; α = 0.74) and “irritable,” “alone,” “angry,” “depressed,” “vulnerable,” “ashamed,” and “anxious” for negative affect (NA; α = 0.89). Subjective Stress was rated by participants on a 10-point scale with the item “Now, I evaluate my stress at…” (0 = No stress to 9 = Extremely stressed) (75). Aggregated mean scores were computed as individual traits for subjective stress. Positive affect (PA) was computed as mean scores of the items “confident” and “happy,” and then aggregated for a PA trait score. Negative affect (NA) was computed as mean scores of the items “irritable,” “alone,” “angry,” “depressed,” “vulnerable,” and “anxious,” and then aggregated for an NA trait score.

#### The Fribourg Reward Task

**Figure 1:**
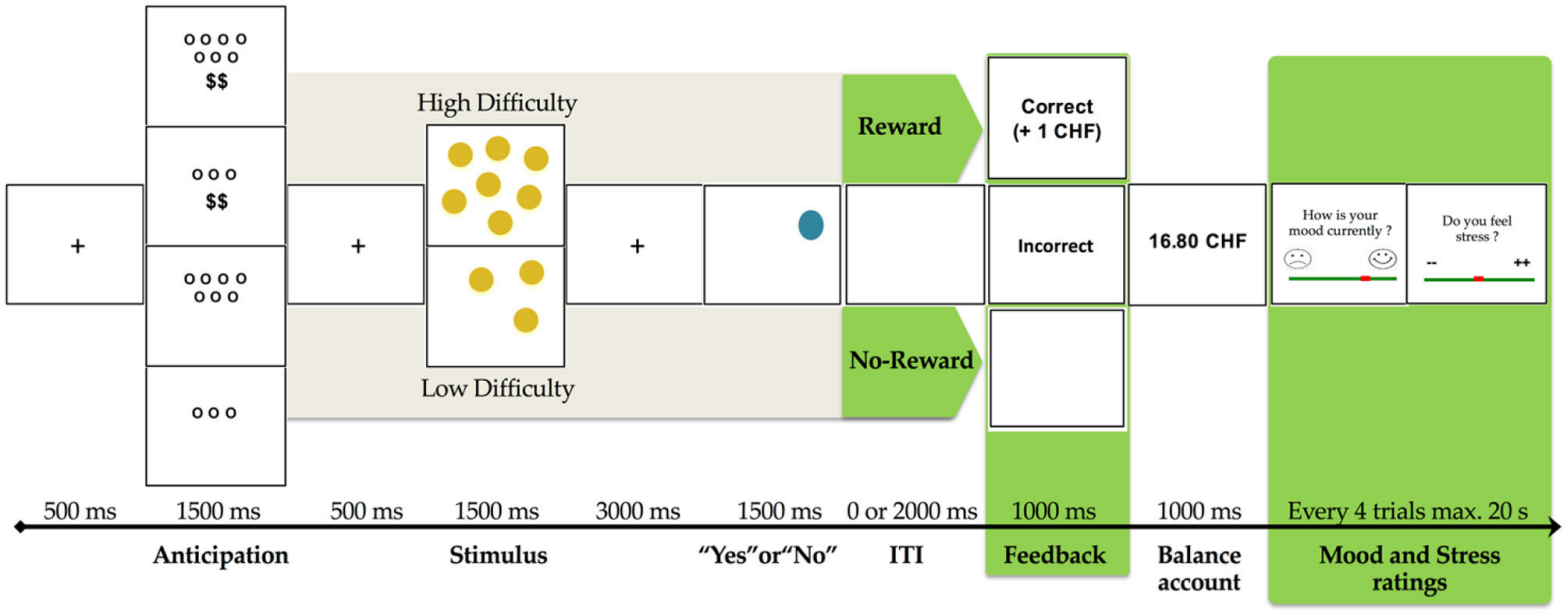
Fribourg reward task. Illustration of trial conditions randomly distributed in both control and stress conditions (unpredictable threat of shock). Variables used in the present study are in green [adapted from Gaillard et al., (76), p. 4].

The Fribourg Reward Task is a monetary incentive delayed task, that was previously shown to elicit striatal activation (36). Participants performed a spatial delayed recall task with two levels of cognitive load (low = 3 circles and high = 7 circles) differentiated by the number of circles to be remembered (see Figure 1). At the onset of each trial, a visual cue showed the level of cognitive load and the monetary reward associated with performance (“blank screen” = no reward or “$$” = reward). Participants then saw a fixation cross (500 ms), followed by an array of yellow circles (3 or 7 circles) (1,500 ms). A fixation cross was then displayed (3,000 ms) before the presentation of the target blue circle, which appeared at any position on the screen during 1,500 ms. With a response box in their right hand, participants responded “yes” or “no” to the question of whether this blue circle occupied a position previously occupied by yellow circles, and did so as quickly as possible. Participants had a maximum of 1,500 ms to respond. After that, a blank screen was displayed during a variable jittered inter-stimulus-interval (ISI; 0 or 2,000 ms) and the feedback displayed (1,000 ms) “blank screen” for no reward or “1 CHF” for reward gain. A final display (1,000 ms) showed a blank screen or the “accumulated amount of gain.” Every four trials, participants rated their mood and stress levels (max. 20 s). Task-related mood and stress were rated by participants on a 10-point Likert scale (0 = Emoticon with very negative mood and 9 = Emoticon with very positive mood), as was current stress (0 = “- -” No stress and 9 = “++” Extremely stressed), all within a maximum of 20 s (see Figure 1).

Correct responses were rewarded in the reward condition (“$$”), but not in the no-reward condition (“blank screen”). Each participant performed two distinct block sessions. In the second block, we added an experimental stress condition with six unpredictable mild electric shocks, previously adjusted to the participant's level of sensitivity. At the beginning of the second block, participants were informed that they would receive electrical shocks unrelated to the task and that they might receive electrical shocks at any time during the block. Before entering the scanner, every participant practiced the task to ensure a good understanding of it and answered questions. The task was implemented using E-Prime Professional (Version 2.0.10.353, Psychology Software Tools, Inc.). Stimuli were presented via goggles (VisualStimDigital MR- compatible video goggles; Resonance Technology Inc., Northridge, CA, USA) with a visual angle of 60°, a resolution of 800 × 600 pixels, and a 60 Hz refresh rate. In this current study, we considered only the reward (reward vs. no-reward) factor of the experiment in our analyses to test our a priori hypotheses.

#### Acute Experimental Stress Manipulation

We induced an acute stress condition in participants during the second block of our experimental design with an unpredictable mild electric shock on the external side of the left hand. The electrical shock intensity was calibrated to each participant before they entered the scanner with a standard shock workup procedure, starting at the lowest level and increasing the intensity until the participant identified an “aversive, but not painful” feeling (77). Electric shocks were induced through an electrical pain stimulator using the PsychLab^©^ measuring system, with MRI-compatible electrodes and cables. The highest allowable shock intensity level was 5 mA (milliamperes).

#### MRI Data Acquisition

Magnetic resonance imagery (MRI) was performed at the Department of Diagnostic and Interventional Neuroradiology of the University Hospital of Bern, Switzerland. The functional MRI images were acquired using a Siemens (Erlangen, Germany) TrioTim syngo 3.0-Tesla whole-body scanner equipped with a radio frequency 32-channel head coil. MRI acquisition included 3D T1-weighted (Magnetization Prepared Rapid Acquisition Gradient Echo; MPRAGE) images with the following settings: sagittal slices: 176; FOV: 256 × 256 mm; matrix size: 256 × 256; voxel size: 1.0 × 1.0 × 1.0 mm^3^; TR: 1,950 ms; TE: 2.2 ms; flip angle: 90°. The event-related task-based fMRI included an EPI pulse sequence with the following settings: interleaved ascending slices: 38; FOV: 230 × 230 mm^2^; matrix size: 64 × 64; voxel size: 3.6 × 3.6 × 3 mm^3^; TR: 2,000 ms; TE: 30 ms; flip angle: 90°.

#### fMRI Data Analysis

fMRI data were analyzed using Statistical Parametric Mapping software (SPM12; https://www.fil.ion.ucl.ac.uk/spm/). The echo-planar images were realigned to the 37th volume, slice timing corrected, coregistered to the structural MR image, spatially normalized to standard Montreal Neurological Institute (MNI) 152 coordinate space, resampled into 3 × 3 × 3 mm voxels, and smoothed with an isotropic 6-mm full-width half maximum Gaussian kernel. Statistical analysis was performed within the framework of the general linear model. We considered only the reward delivery phase as robust striatal differences between FHN and healthy have been evidenced in this phase in particular (27): Because the main focus of this article was on the relationship between neural activation and ESM measures, we focused our analyses on a specific contrast (reward vs. no reward during the reward feedback phase) to limit the number of analyses, in particular with respect to the small sample sizeFor this reason, we will report here only the results related to the whole brain and ROI analyses in response to reward during reward feedback and their association with the ESM measures. Other data related to this study and this sample have been reported elsewhere, in particular the results related to the anticipation phase (76). For each participant, four distinct events were modeled as separate regressors in an event-related manner for the duration of each phase: (a) trial cue (2,000 ms); (b) stimulus presentation (6,000 ms); (c) feedback (2,000 ms); and (d) mood and stress rating (20,000 ms). Subsequently, these regressors were convolved with the canonical hemodynamic response function implemented in SPM12. The six movement parameters (three translations and three rotations) obtained from the realignment procedure were also included in the model. We used a high-pass filter with a cut-off frequency of 1/128Hz. Only trials with correct responses were analyzed. Statistical analyses of single-subject fMRI data were implemented using a general linear model (GLM) with a total of 20 regressors corresponding to six movement parameters and conditions—Stress (control/Stress) × Load (high/low) × Reward (no/rewarded)—across the four events. Note that only high-reward vs. not rewarded trials were used in analysis to increase contrast. A second-level (random-effects) model analysis was performed with independent *t*-test for group analyses. Contrast maps were constructed for the main effect of Reward (high reward > not rewarded), Stress (no-stress vs. stress), and Load (high vs. low), as well as interaction effect for Reward × Stress, Reward × Load, and Stress × Load, for both anticipation and feedback delivery phases. These contrast maps were used for both region of interest (ROI)-based statistical analyses and for whole-brain main effects analysis. For ROI-based analyses, a mask was created with automated anatomical labeling (AAL2) template (78, 79) for bilateral caudate, putamen, and pallidum regions, with two added parcellations for the bilateral nucleus accumbens (Nacc) to create a mask of striatal regions typically involved in reward processing based on (16). An alpha of 0.05 was used with correction for multiple non-independent comparisons using Gaussian random field theory (80) and suprathreshold cluster-size statistics (81). The initial voxel-level threshold for all analyses was set at *p* < 0.001, uncorrected. We used conservative whole-brain correction and kept clusters that reached significance after Family Wise Correction (FWC) at *p* < 0.05. Parameter estimates (beta weight) were extracted from coordinates that showed significant activation after FWC at *p* < 0.05, based on the average activation within the ROI using the MarsBaR toolbox (http://marsbar.sourceforge.net), and referred based on the AAL2 atlas (78, 79) for the main effect of Reward (i.e,. reward condition vs. no reward condition) during the outcome phase in the control condition and in the stress condition.

To control the effects of the reward task, we performed a 2 × 2 × 2 × 2 repeated measures ANOVA including Group (FHD vs. HC) as the between-subject factor, and Stress (no- vs. threat-of-shock), Reward (no- vs. reward), and Load (high vs. low) as within-subject factors for responses accuracy, reaction times (RT) and self-reported mood and stress scores during the task. Results were adjusted with Bonferroni correction for multiple comparisons. We expected faster RT and higher accuracy, higher mood scores during reward as well as an effect of stress on these variables. In particular, we expected higher self-reported stress scores during the stress condition.

Correlations with ESM measures were performed using the beta-weights obtained for the contrast of interest and the self-reported mean for PA, NA and subjective stress over 7 days. We used SPSS (IBM SPSS Statistics, Version 25.0, Armonk, NY, USA) for descriptive analyses of the participants, independent *t*-test and χ^2^ analysis.

## Results

### Participants

Socio-demographic and clinical description of the participants is presented in Table 1. The FHD did not differ significantly from the HC in terms of gender, age, or socioeconomic status. Both groups were mainly composed of students (87 and 81%, respectively). The results of semi-structured interview for depressive symptoms MADRS (68), as well as self-reports for depressive symptom severity BDI-II (69) and for state and trait anxiety (STAI) (82) did not differ significantly between FHD and HC groups. In both groups, one participant has reached BDI-II (69) scores above the clinical threshold. This was not the case for the MADRS (68) scores.

Our results showed that 44% reported currently living with the parent with the history of MDD. Nearly all participants (94%) had lived with their depressive parent. Parents with a history of MDD were mainly mothers (75%); one participant reported that both parents had a history of MDD.

### Behavioral Data Analyses

Table 2 presents the detailed results for the behavioral data analyses for the task.

**Table 2:** Main and interaction effects for within- and between-subject contrasts on behavioral responses related to Fribourg reward task performance during fMRI.

				**RT**			**Accuracy**			**Mood**			**Stress**		
**Within-subject contrasts**	**Stress**	**Reward**	**Load**	***F*_**(1, 30)**_**	***p***	**η^2^**	***F*_**(1, 30)**_**	***p***	**η^2^**	***F*_**(1, 30)**_**	***p***	**η^2^**	***F*_**(1, 30)**_**	***p***	**η^2^**
Stress	Stress vs. control			**17.38**	**<0.001**	**0.37**	**7.14**	**0.01**	**0.19**	**3.93**	**0.06**	**0.12**	2.19	0.15	0.07
Reward		R vs. NR		0.96	0.33	0.03	**3.98**	**0.05**	**0.12**	**4.01**	**0.05**	**0.12**	0.32	0.58	0.01
Load			H vs. L	**130.94**	**<0.001**	**0.81**	**84.23**	**<0.001**	**0.74**	2.14	0.15	0.07	0.18	0.67	0.01
Stress × group	Stress vs. control			0.17	0.68	0.01	2.09	0.16	0.06	0.08	0.77	0.00	0.04	0.85	0.00
Reward × group		R vs. NR		0.14	0.71	0.00	0.90	0.35	0.03	0.18	0.67	0.01	0.22	0.64	0.01
Load × group			H vs. L	1.27	0.27	0.04	0.80	0.38	0.03	1.88	0.18	0.06	0.01	0.91	0.00
Stress × reward	Stress vs. control	R vs. NR		2.44	0.13	0.07	0.94	0.34	0.03	0.04	0.85	0.00	0.12	0.73	0.00
Stress × load	Stress vs. control		H vs. L	3.14	0.09	0.09	0.10	0.75	0.00	0.06	0.80	0.00	0.09	0.76	0.00
Reward × load		R vs. NR	H vs. L	0.44	0.51	0.01	**5.65**	**0.02**	**0.16**	0.15	0.70	0.00	0.74	0.40	0.02
Stress × reward × group	Stress vs. control	R vs. NR		0.14	0.71	0.00	1.90	0.18	0.06	0.02	0.88	0.00	0.07	0.79	0.00
Stress × load × group	Stress vs. control		H vs. L	3.23	0.08	0.10	0.43	0.52	0.01	0.64	0.43	0.02	0.16	0.69	0.00
Reward × load × group		R vs. NR	H vs. L	2.45	0.13	0.08	0.06	0.81	0.00	0.02	0.89	0.00	0.06	0.80	0.00
Stress × reward × load	Stress vs. control	R vs. NR	H vs. L	0.00	0.97	0.00	0.62	0.44	0.02	1.05	0.31	0.03	1.14	0.29	0.04
Stress × reward × load × group	Stress vs. control	R vs. NR	H vs. L	3.07	0.09	0.09	2.94	0.10	0.09	0.36	0.55	0.01	0.15	0.70	0.00
Between-subject contrasts	Group														
Group	FHD vs. HC			2.22	0.15	0.07	0.04	0.84	0.00	0.44	0.51	0.01	0.02	0.89	0.00

*Results are corrected for multiple comparisons by applying a Bonferroni correction. Bold indicates two-tailed (p < 0.05) and one-tailed (p < 0.05/2) significant results. RT, Reaction time; R, Rewarded; NR, Not rewarded; H, High; L, Low. Partial eta squared (η^2^) values range from 0 to 1, and represents the proportion of total variance accounted for by the factor(s), while excluding other factors from the total explained variance (i.e., non-error variation) in the repeated measures ANOVA (83)*.

#### Reaction Time and Accuracy

For RT, we found significant main effects for the Stress [*F*_(1, 30)_ = 17.38, *p* < 0.001, η^2^ = 0.37], and Load conditions [*F*_(1, 30)_ = 130.94, *p* < 0.001, η^2^ = 0.81] as well as a statistical trend for the Stress x Load interaction [*F*_(1, 30)_ = 3.23, *p* = 0.08, η^2^ = 0.10]. *Post-hoc* tests indicate that FHD and HC individuals were responding faster in the stress condition (*M* = 730.66 ms, *SE* = 16.60 ms) than in the control condition (*M* = 784.34 ms, *SE* = 16.36 ms); as well as faster in the high load condition (*M* = 709.35 ms, *SE* = 15.77 ms) than in the low-load condition (*M* = 805.65 ms, *SE* = 15.73 ms). We did not consider further the statistical interaction stress × load as this is not the main focus of the current study.

For accuracy, we found significant main effects for the Stress [*F*_(1, 30)_ = 7.14, *p* < 0.01, η^2^ = 0.19], Reward [*F*_(1, 30)_ = 3.98, *p* < 0.05, η^2^ = 0.12] and Load [*F*_(1, 30)_ = 84.23, *p* < 0.001, η^2^= 0.74] factors as well as a significant interaction reward × load [*F*_(1, 30)_ = 5.65, *p* < 0.02, η^2^ = 0.16]. Both FHD and HC individuals provided more accurate responses in the stress conditions (*M* = 82.6%, *SE* = 2.1%) than in the control (no-stress) conditions (*M* = 78%, *SE* = 2.1%), in the low load condition (*M* = 87.3%, *SE* = 2%) than in the high load condition (*M* = 73.3%, *SE* = 2.1%), and in the reward condition (*M* = 81.6%, *SE* = 1.9%) than no-reward condition (*M* = 79%, *SE* = 2.2%). Both FHD and HC individuals provided more accurate responses for high load in the reward condition (*M* = 76%, *SE* = 2.3%) than in the no-reward condition (*M* = 70.5%, *SE* = 2.5%), while we found no significant increment in the low load condition between the reward (*M* = 87.2%, *SE* = 2%) and the no-reward conditions (*M* = 87.5%, *SE* = 2.2%). No significant group differences were found for RT and accuracy.

#### Self-Reported Mood and Stress

For the self-reported mood scores, our results show significant main effects of the Reward factor [*F*_(1, 30)_ = 4.01, *p* < 0.05, η^2^ = 0.12] factors; and a statistical trend for the Stress factor [*F*_(1, 30)_ = 3.93, *p* < 0.06, η^2^ = 0.12]. *Post-hoc* tests indicate that both FHD and HC individuals rated their mood higher in the reward condition (*M* = 6.87, *SE* = 0.28) than in the no-reward condition (*M* = 6.72, *SE* = 0.28), and in the control condition (no-stress) (*M* = 6.91, *SE* = 0.29) than in the stress condition (*M* = 6.67, *SE* = 0.29). With regard to the stress ratings, we did not find any significant results.

### ESM Protocol: Group Comparisons

Aggregated means and standard deviation of the daily life measurements are reported in Table 1. Results of the PA and NA mean score comparison between the FHD and HC groups showed no significant differences (*p* = 0.74 and 0.78 respectively). Similarly, no group difference was found for the reported daily life stress (*p* = 0.69).

### fMRI Results

Table 3 presents the results of the whole-brain analyses in the contrast of interest. To control for the effect of the stress condition, we also report the regions activated in the main contrast comparing the stress vs. no stress condition.

**Table 3:** Significant BOLD responses to reward delivery in the reward vs. in participants with family history of depression (FHD) and in healthy control (HC).

**Group contrasts**	**Regions**	**L/R**	**MNI coordinates**			**Cluster size**	***T*-value**	**pFWE**
			**X**	**Y**	**Z**			
**Main effect of reward: reward condition** **>** **no reward condition**								
HC > FHD	No significant activation							
HD > HC	Putamen	L	−15	9	0	24	4.77	<0.005
		L	−24	6	−3			
		L	−15	9	6			
ROI								
HC	Caudate	L	−9	−6	0	37	6.21	*P* < 0.001
			−6	0	9		4.80	
FHD	Caudate	L	−15	−6	15	12	6.34	*P* < 0.01
HC	Inferior occipital gyrus	L	−36	−87	−12	3,343	11.52	<0.001
	Inferior occipital gyrus	R	33	−93	−3		10.01	
	Middle occipital gyrus	R	36	−93	6		10.16	
	Superior parietal gyrus	R	36	60	57	470	9.35	<0.001
	Inferior parietal gyrus	R	48	−45	48		8.66	
			30	−54	45		7.89	
	Anterior cingulate cortex	L	−3	33	30	380	7.63	<0.001
			−3	39	15		7.00	
		R	6	39	9		7.34	
	Middle cingulate cortex	R	6	−12	27	77	6.53	<0.001
		R	6	−27	36		5.72	
	Precentral gyrus	L	−51	12	33	192	8.19	<0.001
			−54	6	39		6.17	
	Inferior frontal gyrus	L	−51	33	21		7.22	
	Inferior frontal gyrus	R	54	12	21	189	6.95	<0.001
		R	51	30	18		6.33	
	Middle frontal gyrus	R	45	39	15		5.93	
		R	39	60	−6		6.18	
	Middle frontal gyrus	R	36	54	9	83	6.18	<0.001
		R	45	51	−3		5.89	
	Superior frontal gyrus, dorsolateral	R	36	54	9		6.00	
	Insula	L	−36	9	−12	104	6.8	<0.001
			−42	18	−9		5.48	
	Orbitofrontal cortex	L	−54	27	−6		5.13	
	Ventral tegmental area		0	−15	−9	76	6.06	<0.001
	Periaquaductal area	R	6	−30	−6		5.41	
	Thalamus	R	6	−24	0		5.36	
	Hippocampus	R	21	−27	−9	38	5.63	<0.005
			18	−39	6		5.44	
	Cerebellum	L	−3	−54	−42	53	5.23	<0.001
		L	−15	−57	−36		5.15	
FHD	Fusiform gyrus	L	−33	−57	15	4,640	17.46	<0.001
	Inferior occipital gyrus	L	−30	−90	−9		14.79	
		L	−42	−69	−12		14.54	
	Anterior cingulate gyrus	R	9	33	27	1,010	8.49	<0.001
		L	−3	36	12		8.46	
		L	−3	27	27		7.47	
	Insula	L	−36	18	6	655	9.44	<0.001
		L	−39	9	−12		7.67	
	Orbitofrontal cortex	R	39	33	−3	758	7.74	<0.001
	Insula	R	30	21	−12		7.4	
	Inferior frontal gyrus	R	39	12	30		7.36	
	Inferior parietal gyrus	R	54	−42	48	218	7.6	<0.001
	Superior parietal gyrus	R	54	−33	57		6.43	
	Inferior parietal gyrus	L	−48	−45	45	86	7.02	<0.001
	Post-central gyrus	L	−42	−33	51		5.13	
	Angular gyrus	R	30	−69	48	39	5.37	<0.001
	Superior parietal gyrus	R	33	−60	54		4.80	
	Middle frontal gyrus	R	48	51	12	25	4.93	<0.001

#### Striatal Activation During Feedback: Group Comparison

**Figure 2:**
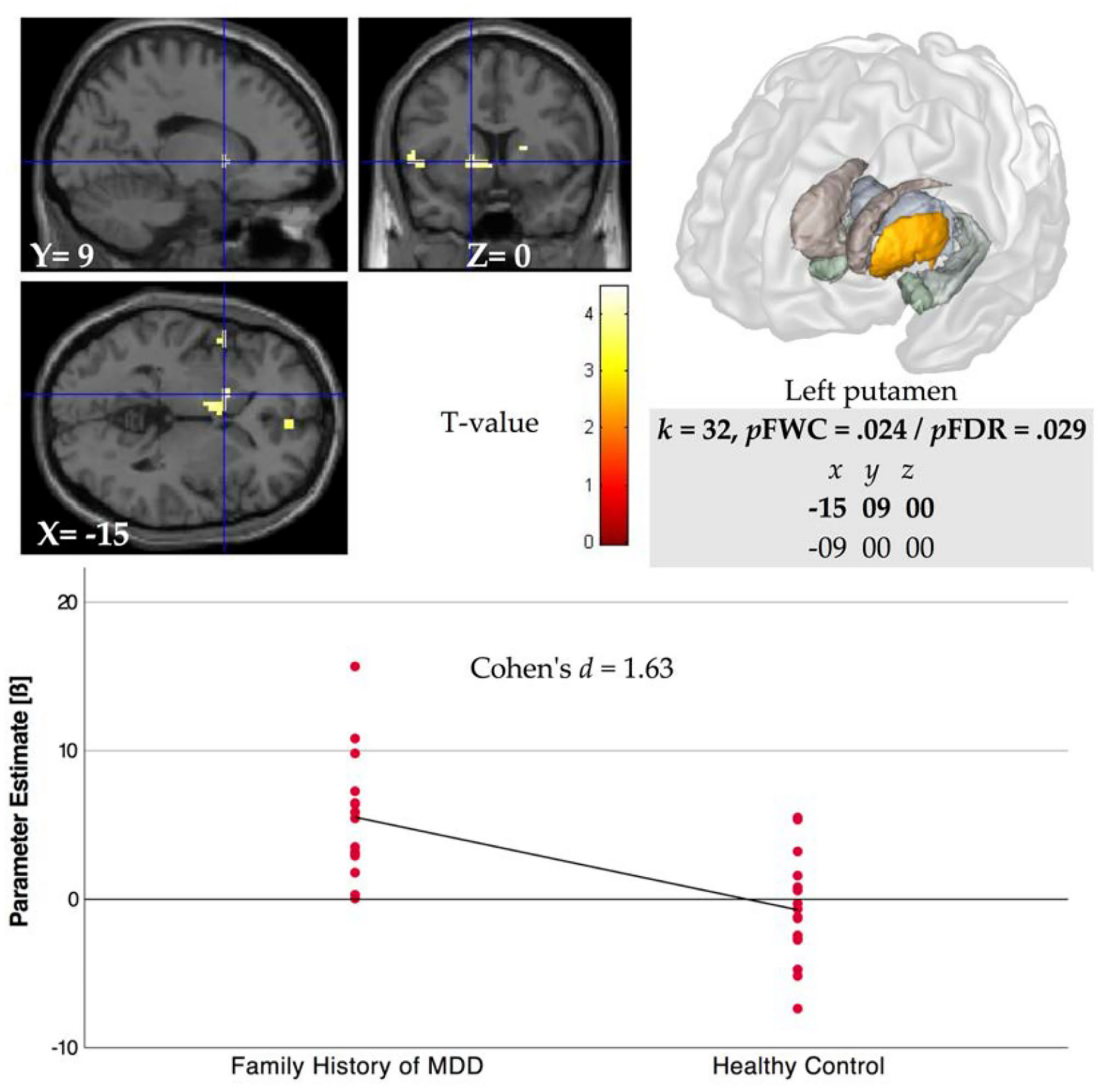
Left ventral striatal (VS, i.e., putamen) region BOLD activation for comparison of FHD and HC groups during reward feedback in stress condition for contrast rewarded > not rewarded (*p* < 0.005 FWE). Parameter estimates (beta weight) were extracted from coordinates that showed significant activation after FWE at *p* < 0.05 in the ROI analyses for the main effect of reward.

The whole-brain analysis for group comparison showed a significant difference in BOLD response in part of the VS, i.e., in the left putamen region between FHD and HC group during feedback delivery for the main effect of reward (reward vs. no reward condition in the control condition, see Table 3) at *p* < 0.005 FWE that remains significant in the stress condition, i.e., comparison of reward vs. no reward condition in the stress condition (see Figure 2). Specifically, we found a stronger VS activation in the FHD group (*M* = 5.53, *SD* = 4.06) than in the HC group (*M* = −0.71, *SD* = 3.58), *t*_(30)_ = 4.46, *p* = 0.024, under stress with a very large effect size (Cohen's *d* = 1.63).

#### VS Reward-Response Under Stress Association With ESM

**Figure 3:**
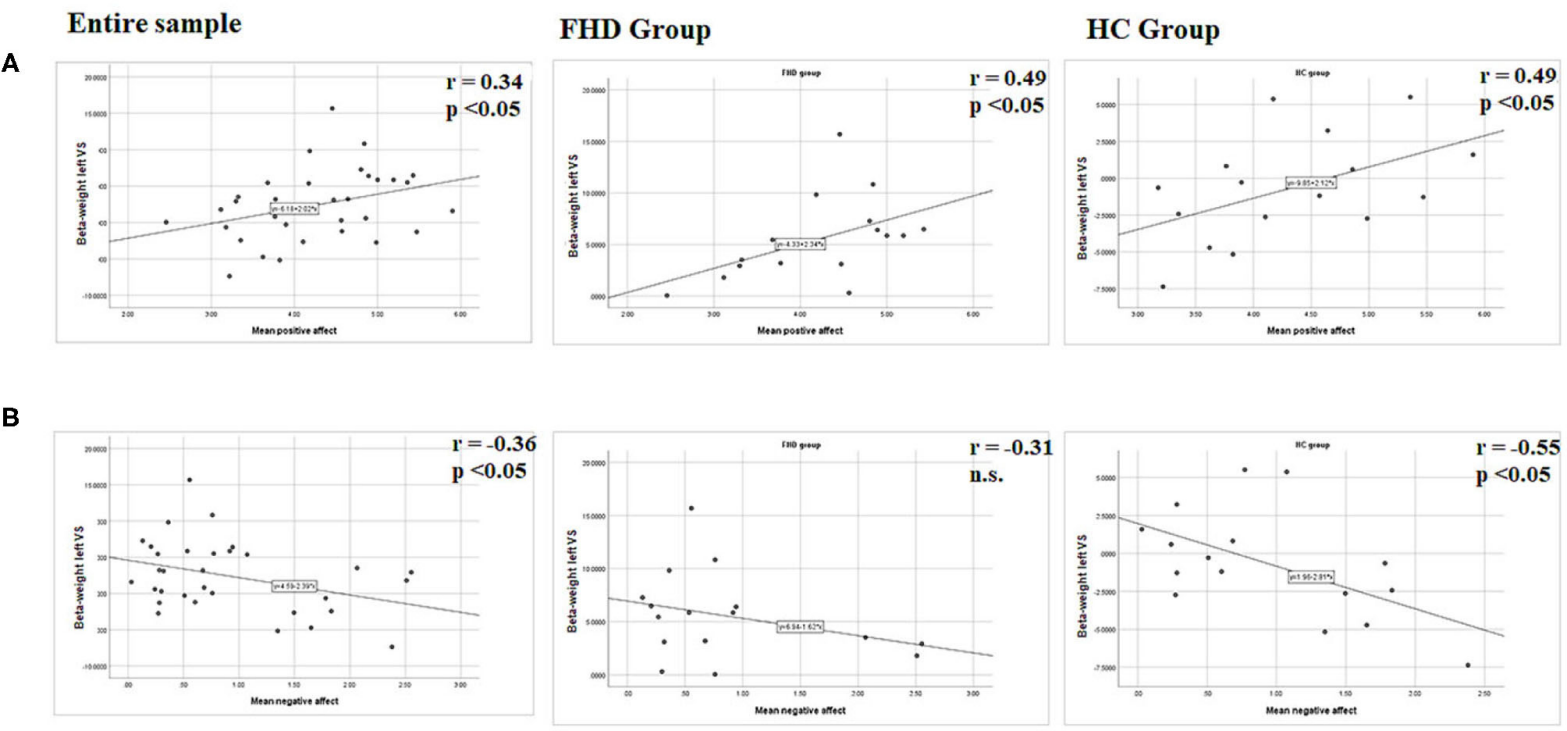
Graphical presentation of the statistical relationships between **(A)** mean positive affect resp. **(B)** Mean negative affect and left ventral striatal (VS) region BOLD activation during reward feedback in stress condition for contrast rewarded > not rewarded. Parameter estimates (beta weight) were extracted from coordinates that showed significant activation after FWC at *p* < 0.05 in the ROI analyses with peak activation in the caudate. Results are presented for the entire group, the FHD group and the HC group. r, Spearmann correlation coefficient, n.s., not significant.

Spearman correlations were performed between beta parameter estimates extracted in the VS based on the striatal mask, whose peak activation was located in the ventral striatum around the left medial caudate (see Table 3) and mean scores of self-reported PA, NA and subjective stress in daily life. Considering both groups together, our results showed a significant positive correlation with PA *r*_s_ = 0.34, *p* = 0.05, and a significant negative correlation with NA *r*_s_ = −0.36, *p* = 0.042 and no significant correlations with reported stress *r*_s_ = −0.21, *p* = 0.22. Considering the groups separately, the positive correlation between VS activation and PA was significant in both groups (FHD: rs = 0.49, *p* = 0.05; HC: rs = 0.49, *p* = 0.05), while the negative correlation with NA was significant only in the HC group (rs = −0.55, *p* = 0.02) and not in the FHD group (rs = −0.31, *p* = 0.23); and the correlation with reported stress remained not significant (FHD: rs = −0.29, *p* = 0.27; HC: rs = −0.13, *p* = 0.62), (see Figure 3).

#### Additional Regions Activated During Feedback

The whole-brain analysis for the main effect of reward showed significant differences in BOLD response in the comparison of the reward condition vs. the no-reward condition bilaterally in the occipital cortex, the anterior cingulate cortex, and the inferior frontal gyrus as well as in the right parietal cortex, right middle cingulate gyrus, right middle and superior frontal gyrus, right periaquaductal area, right thalamus, right hippocampus and in the left insula, left orbitofrontal cortex, and left cerebellum in the HC participants. In the FHD group, we found significant differences in BOLD response bilaterally in the anterior cingulate gyrus, the insula, and the parietal cortex as well as the right orbitofrontal cortex, right middle frontal gyrus and left occipital cortex (see Table 3).

#### Regions Activated in Response to Stress

The whole-brain analysis for the main effect of reward showed significant differences in BOLD response in the comparison of the stress condition vs. the no stress condition in the right superior parietal cortex, right lateral occipital cortex, right precuneus, right caudate as well as in the left superior frontal cortex and left insula in the healthy controls. In the FHV group, our results evidenced bilateral significant differences in BOLD responses in the parietal cortex that were also significantly more activated in the group comparison.

## Discussion

To our knowledge, this may be the first study to report a significantly increased ventral striatal neural response to reward delivery received during stress exposure in individuals with FHD compared to healthy controls. These results are counter to our hypothesis and previous findings on the blunting effect of stress on the hedonic capacity (84–86). Another remarkable finding is the association between the observed ventral striatal activation with daily life measures of PA in FHD participants and healthy participants as well as a significant negative correlation with daily life measures of NA that was significant only in the healthy control group.

Unexpectedly there was no significant difference in the striatal activation during reward delivery between FHD and HC in the condition without stress. This differs from previous findings on blunted striatal responses to reward in high-risk individuals (24–27). This could be related to the lack of power; the sample may have been too small to detect difference between FHD and HC groups. However, McCabe et al. (22) did not report any striatal response to reward difference between groups with high and low risk of MDD. A common factor, shared by our study and McCabe et al.'s (22) previous research, is related to the mean age of the sample, which is older in our study (above 20 years). Striatal development studies have shown an important change between childhood and early adulthood in healthy individuals (87), and individuals with FHD (27). In addition, evidence demonstrates that neural response sensitivity to monetary and social reward changes across developmental stages (88). A further explanation could be related to the design, since participants might have been expecting the stress condition, and the condition without stress cannot be considered without taking into account the stress condition.

The increased sensitivity to reward outcomes during stress exposure for the FHD group compared to the HC group is consistent with a heuristic model of depression and the specific influence of stress on reward processing (9), as well as with psychobiological mechanisms of resilience and vulnerability (89). In our sample, the increased sensitivity to reward in the stress condition could be interpreted as a sign of a specific resilience marker in a brain region (i.e, the putamen) previously related to vulnerability to family risk of MDD (27). Putamen activation has been suggested to play a unique role in the intergenerational risk of depression, with evidence of an association between maternal and daughter putamen responses to anticipation of loss (90). Since we excluded participants with a previous history of mental disorder and since our sample was composed of young adults and not of adolescents, we might have included resilient individual, i.e., individuals who had passed through the high risk phase of adolescence without developing MDD or another psychopathology. This hypothesis is supported by the finding that the groups did not differ with regard to their subjective stress ratings, PA and NA measures in everyday life. Thus, in our results the increased VS response to reward delivery under stress could be a marker of a resilient profile. This interpretation should be however be taken with caution due to the small sample of participants, and because we did not use a longitudinal setting.

In line with that hypothesis, our significant association between increased ventral striatal reward reactivity and PA in daily life could be interpreted as a protective factor. Previous findings showed that the VS response to reward was associated to PA in daily life (35, 91). A higher VS response to winning has been reported as a resilience marker in adolescent girls with unknown parental mental health histories (92). High sensitivity to reward experiences in daily life has been shown to increase resilience after environmental adversity (57). More PA after stress events has been shown to mediate the relation between sensitivity to reward and trait resilience (93). More broadly, increased reward response could buffer and blunt stress responses more quickly in a less predictable environment [for a review of a reward pathway buffering stress; (94) #132]. In that context, our unexpected finding that there was not reduced self-reported reward sensitivity (measured as PA) in the FHD group, could be associated with the hypothesis that we might have included resilient individual, i.e., individuals who did not develop psychopathological problems during the high-risk period of adolescence. An addition to the existing literature comes from our finding of a significant negative correlation between daily life NA and ventral striatal activation to reward that was specific to the HC group. To our knowledge, no study has investigated the correlation between neural reward reaction and NA.

In addition to the results observed in striatal regions, we also found in both groups significant reward-related activations in regions, which have been typically associated with the cerebral reward system (12), including the orbitofrontal and medio-prefrontal cortex and the anterior cingulate gyrus in both groups of participants. Interestingly, our results also evidenced significant reward-related BOLD responses in the occipital and the parietal cortex. This is in line with previous studies showing for instance increased responses in the occipital cortex to rewarded tasks, especially in tasks involving visual attention (95). Activation in the parietal cortex was reported in response to reward tasks, in particular in tasks involving several levels of reward (96) as this is the case in our task. However, we found no significant group difference in any of these regions, but regions of the parietal cortex were also significantly more activated in the stress condition and this activation was also more accentuated in the FHD group than in the HC group. Increased activation in parietal regions in response to acute experimental stress has been documented in previous studies [for instance (97)] and interpreted as an augmented cognitive control under stress conditions. This increased activation in regions associated with cognitive controls could therefore also be associated with the observed better performance during the task (e.g., faster reaction times and increased accuracy) in the stress condition.

Our study has some limitations. First, the small sample size of this preliminary study did not allow us to investigate participants' age in relation to parental onset of MDD, or to use years lived with depressed parents to predict striatal activation. Secondly, our design did not include a counterbalanced condition in the no-stress (control) and stress (unpredictable threat of shock) conditions. In that context, the observed stress main effect in reaction times and accuracy could reflect a learning effect rather than a stress effect. The lack of counter-balancing cannot however explain the lack of group difference in the condition without stress, since the same potential flaw was balanced out in the group comparison. Thirdly, our results did not evidence differences in stress ratings between the control and the stress conditions. This could be related to the small sample size as the results obtained in a larger associated sample could evidence significant stress ratings differences between the conditions (36). In addition, the different levels of cognitive load could have induced stress and be a confounding factor. Fourthly, in both groups of participants, one participant evidenced BDI scores above the clinical threshold. This could indicate that we included participants with increased depressive symptomatology in both groups or this could be related to a misunderstanding of some questions of the BDI-II, since no participant had MADRS scores above the clinical threshold and no participant fulfilled the depression criteria as determined by the MINI (61). Self-report questionnaires tend to overreport and clinician-based measures are thus the gold standard. Fifthly, the fact that a blank screen was presented in the no-reward condition in the feedback phase did not allow us to control for the brain activation related to the processing of the salience, visual attention and reading processes. Sixthly, the observed activation differences between the groups in the putamen were significant at a reduced thershold (*p* < 0.005). Seventhly, using average scores for the ESM data analysis might have obscured some important features of the experience sampling data. Measure of variability might have taken better advantage of the rich dataset and provided a better measure of emotional lability in everyday life. Finally, our results showed only associations, and a prospective design would be needed to enable the accumulation of causal and predictive evidence. Altogether, our results should be taken as preliminary and as a first step toward thinking about new pathways for studying the psychophysiological dynamics of reward processes within the laboratory and daily life environments.

## Conclusion

Our results indicate that an increased family risk of depression was associated with specific striatum reactivity to reward in a stress condition. This is in line with previous studies showing atypical responses to reward in individuals at risk of depression. This finding extends the literature by investigating the stress-reward interaction in these individuals. Our results support previous findings that ventral striatal reward-related response is associated with PA in daily life, (46). A new finding is the negative association between NA in daily life and reward-related ventral striatal activation that was observed in the HC group but not in the FHD participants. Due to the small sample size, these results must be considered preliminary. We suggest that our integrative approach might be a promising way to tackle subtle processes and differences in the field of vulnerability research.

## Data Availability Statement

The raw data supporting the conclusions of this article will be made available by the authors, without undue reservation.

## Ethics Statement

The studies involving human participants were reviewed and approved by commission d'éthique du Canton de Vaud. The patients/participants provided their written informed consent to participate in this study.

## Author Contributions

CM-S, AH, PG, DS, CM-P, and GH contributed to the design of the study. MG, CG, and RR performed the study and the data analysis. CG wrote parts of the manuscript. AF was instrumental for the fMRI set up and data preparation. PH provided the electro shock methodology and stress application. CM-S and MG wrote conjointly the manuscript. All authors contributed to the manuscript revision, and read and approved the submitted version.

## Conflict of Interest

The authors declare that the research was conducted in the absence of any commercial or financial relationships that could be construed as a potential conflict of interest.
